# Comprehensive Quantitative Spatiotemporal Gait Analysis Identifies Gait Characteristics for Early Dementia Subtyping in Community Dwelling Older Adults

**DOI:** 10.3389/fneur.2019.00313

**Published:** 2019-04-05

**Authors:** Anne-Marie De Cock, Erik Fransen, Stany Perkisas, Veronique Verhoeven, Olivier Beauchet, Maurits Vandewoude, Roy Remmen

**Affiliations:** ^1^Department of Geriatrics, University of Antwerp, Antwerp, Belgium; ^2^Department of Primary and Interdisciplinary Care (ELIZA), University of Antwerp, Antwerp, Belgium; ^3^Department of Geriatric Medicine, General Hospital ZNA, Antwerp, Belgium; ^4^StatUa Centre for Statistics, University of Antwerp, Antwerp, Belgium; ^5^Division of Geriatric Medicine, Department of Medicine, Centre of Excellence on Aging and Chronic Disease (CEViMaC), McGill University, Montreal, QC, Canada

**Keywords:** comprehensive spatiotemporal gait analysis, motor cognitive risk, dementia subtype, screening, older adults

## Abstract

**Background:** Recent studies associated gait patterns with cognitive impairment stages. The current study examined the relation between dementia type and spatiotemporal gait characteristics under different walking conditions in pre and mild neurocognitive disorder stage.

**Methods:** Community-dwelling older adults (age 50+) with memory complaints consulting a memory clinic underwent, at baseline and during follow-up (every 4 months), a standard dementia assessment and a comprehensive spatiotemporal gait analysis [walking on an electronic walkway at usual pace (UP) with and without a counting-backwards (CW) or animal-reciting dual-task (AW), at fast (FP) and at slow (SP) pace]. At baseline the participants were categorized according to the Clinical Dementia Rating (CDR) scale. At the end of the study, the dementia diagnosis was used to stratify the categories in three outcome groups: developed “No-dementia,” “AD+FTD” (grouping Alzheimer's or Fronto-temporal dementia) or “VascD+LBD” dementia (grouping Vascular dementia or Lewy body dementia). The gait characteristics were compared per category in paired groups. Sub-analyzing in the ≥70-years-old participants evaluated the age effect.

**Results:** Five hundred and thirty-six participants, age 50-to-95-years old were followed for 31-to-41 months. In the CDR 0, no differences were seen between eventual dementia and no-dementia individuals. In the CDR 0.5, CW dual task cost (DTC) step width was larger in the imminent “AD+FTD” and AW (normalized) gait speed was slower in the future “VascD+LBD” group compared to the no-dementia participants. Slower UP (normalized) gait speed differed the future “VascD+LBD” from the “AD+FTD” individuals. In the CDR 1: Wider steps in UP, SP and CW differed the “VascD+LBD” from the “AD+FTD” group. In the ≥70-years old CDR 0 category, higher AW cycle time variability in the imminent “AD+FTD” dementia group, wider UP step width and higher AW cycle time variability in the “VascD+LBD” group differed them from the no-dementia group up to 3 years before dementia diagnosis. The distinctive gait characteristics between the no-dementia and the imminent dementia groups in CDR 0.5 and CDR 1 remained the same as in the overall group. However, no gait differences were found between “VascD+LBD” and “AD+FTD” groups in the pre-dementia stages.

**Conclusion:** Distinctive spatiotemporal gait characteristics were associated with specific dementia types up to 3 years before diagnosis. The association is influenced by the cognitive stage and age.

## Introduction

The most recent diagnostic criteria for clinical diagnosis of dementia types in preclinical and dementia stages encompass clinical features, diagnostic imaging, neurocognitive testing techniques and biomarkers to classify patients with Alzheimer's, Frontal lobe, Lewy body or Vascular dementia (1–6). So far, mobility features have not been used as diagnostic criteria. However, gait instability and falls are frequently seen in these disorders, and gait analysis may provide addition features to distinguish between them (7–9).

Spatiotemporal gait characteristics (SGCs) have recently been associated with the stages of cognitive impairment (10). The Comprehensive Spatiotemporal Gait Analysis in different walking conditions can predict the presence of cognitive impairment stages. Also, the Gait, cOgnitiOn & Decline (GOOD) cross-sectional study on gait phenotype in individuals with cognitive impairment demonstrated that specific profiles of gait impairment were related to the stage and the type of cognitive impairment (11).

Using gait characteristics to distinguish between the dementia types, however, is still a topic of discussion. This dementia subtyping between Alzheimer's dementia (AD) patients and adults with any dementia was suggested in stride-time and stride-width variability (12, 13). The distinction between AD and individuals with subcortical lesions seemed related to gait speed (14). The GOOD initiative identified that gait characteristics in non-amnestic MCI participants are more altered than in amnestic MCI subjects. Furthermore, the quality of gait was better than in the individuals with Alzheimer dementia (AD). Non-AD participants, by contrast, presented with worse gait performance then those with AD (15). Also, a study on the Motor Cognitive Risk syndrome (MCR) subtypes suggested that even in the pre-clinical dementia stage (16), specific features of gait could be linked to different cognitive profiles in individuals characterized by slow gait and cognitive complaints. A meta-analysis comparing people with neurological disorders to healthy individuals of the same age, on the other hand, concluded that gait variability and altered gait dynamics showed no distinction between the types of neurocognitive disorders (17). Research on Spatiotemporal Gait Analysis in Lewy body dementia (DLB) and AD showed that single-task walking changed similarly in the AD and DLB in comparison with non-demented (cognitively normal) persons as velocity and stride length decreased and double support (two feet simultaneously touching the floor) increased in both groups (18).

A recent review on the use of gait analysis in dementia subtyping, however, suggested that research on more comprehensive quantitative gait analysis was needed to address this issue (19). Also a review on the discriminating capacity of Comprehensive Gait Assessment including instrumented gait analysis and balance assessment concluded that dual tasking could be useful in the differentiation between mild cognitively impaired and cognitively intact individuals (20).

According to this view, investigating the gait characteristics in different walking conditions in preclinical and mild cognitive impaired individuals prospectively would create the opportunity to discover associations between early gait characteristics and a future dementia diagnosis. Furthermore, characterizing those at-risk of a dementia subtype might be helpful for early treatment and preventive actions. Moreover, the early typing of individuals in the pre-dementia stage could enable clinicians to anticipate the problems and the risk factors of the specific dementia subtype. In addition, typing could help identify specific risk groups for more targeted research.

In this present study, we hypothesize that early changes in spatiotemporal gait characteristics in different walking conditions were associated with the risk for developing a specific dementia type within a period of 5 years in older adults with cognitive complaints, mild cognitive impairment and mild dementia. The objectives were to identify the differences in the gait parameters, to describe the added value of a multiple walking conditions protocol and examine the impact of age on these parameters.

## Patients and Methods

### Participant Selection Criteria, Physical Assessment, and Questionnaires

All community dwelling individuals older than age 50 years attending a Memory Diagnosis Centre were eligible for inclusion. The participants had to be still living at home. Mini Mental State Examination (MMSE) was used as cognitive screening at inclusion (21). Participants with MMSE >10 were enrolled in the study. Patients were entered consecutively in a database between 2010 and 2015. The study was part of a larger project, investigating the use of quantitative gait analysis for cognitive screening and diagnosis. In previous work, the methodology for participant selection, criteria and assessment were described (10).

The demographic status, medical history, social status, care support and Rockwood's frailty index (22) were registered. These data included: age, gender, ethnic background, education level, disability using Activity of Daily Living (ADL or Katz) scoring (23) and instrumental ADL (iADL) function evaluated according to Lawton/Brody (24), the number of medications and Timed Get Up and Go test (tGUG) (25, 26). Fall risk was assessed using three parameters: by asking the question: “did you fall during the last 12 months,” the Timed Chair Stand test (TCST) as part of the Short Physical Performance Battery (27) and the Functional Reach test (FR) (28). The tGUG test, TCST and FR test are validated tests used in geriatric examination and considered good practice in the geriatric assessment for falls. These tests should be performed in stable clinical conditions (29–32).

From participants' medical records, we obtained information on the presence of depression, cardiac ischemia, heart failure, hypertension, cerebrovascular ischemia, diabetes, chronic obstructive lung disease, and gait disorders (Parkinson's disease, parkinsonism or arthritis).

At baseline, the participants were categorized in five groups based on the Clinical Dementia Rating (CDR) scale levels (33, 34): the Cognitively Healthy Individuals (CHI = CDR 0), the Mild Cognitively Impaired (MCI = CDR 0.5), the mild (CDR 1), moderate (CDR 2) and severe dementia (CDR 3) patients. Cognitively healthy individuals were considered as such when clinical diagnostic criteria for cognitive dysfunction were not met at baseline. This group could include normal adults and individuals in a preclinical dementia stage. All analyses were carried out for all five CDR groups. This study report focused on CHI, MCI and mild dementia because these phases of cognitive complaint or impairment were the most clinically relevant for diagnosis and early treatment planning (35).

The dementia diagnosis was performed after cognitive screening and inclusion. MMSE and “Addenbrook's cognitive evaluation- revised” translated in Dutch (ACE-R) were used for general cognitive screening. The combination of these two screening methods has good accuracy, clinical utility and is currently accepted as good practice in screening for cognitive decline in clinical settings. The combination also provides information on the cognitive domains and differentiation whether or not cognitive impairment was present (36–38). A geriatrician and a neurologist performed a geriatric assessment and a physical and neurological examination. Table S1 summarizes the demographic parameters, cognitive diagnostic tests and questionnaires, physical parameters, scales and disability assessment scores used in this assessment. Brain imaging using Multi-slice Computer Tomography was performed in all included participants and coded for the presence of white matter lesions (yes or no) and the region of cortical atrophy (none, frontal, parietal/temporal, global) by one geriatrician and one radiologist. When clinically indicated, a fludeoxyglucose (^18^F)—positron emission tomography (FDG-PET) scan was performed to refine the diagnosis. The WAIS-IV neuropsychological battery was used to complete the analysis and to establish the dementia diagnosis according to the National Institute on Aging and the Alzheimer's Association workgroup revised criteria for Alzheimer's disease, dementia types and pre-dementia stages and clinical guidelines for Lewy body disease, Vascular and Fronto-temporal lobe dementia (1–6). Using these criteria, we defined six neurocognitive disorder outcome groups: MCI (incorporating amnestic mild cognitively impaired (aMCI), non-amnestic mild cognitively impaired (naMCI) and multi-domain MCI), Alzheimer type dementia (AD), mixed/vascular dementia (VascD), Lewy body Dementia (LBD) and Fronto-temporal lobe dementia (FTD) patients next to “normal” individuals, who had no dementia at the closing of the study.

All included participants were re-evaluated in a longitudinal follow-up every 4 months. During the follow-up period up to 5 years: gait analysis, cognitive testing, dementia typing, social status, and disability screening were recorded. The dementia diagnoses at the end of the study period were used for the stratification. This allowed us to identify how the cognitively “normal” individuals with cognitive complaints and mild cognitively impaired evolved within the 5-years study period.

### Technical Investigation

#### Spatiotemporal Gait Analysis

Gait analysis was performed on a *GAITRite*^®^ *platinum* electronic walkway *(CIR systems, Havertown, PA, USA)*. We used a comprehensive spatiotemporal gait assessment method, namely “the 5-walk-test method” as described in previous work (10). Gait analysis was performed on a 6.1 meter computerized walkway with embedded pressure sensors (GAITRite® platinum, CIR systems, Havertown, PA, USA) permanently installed in a test room equipped in agreement with the GAITRite® users group criteria in a quiet, indirectly lit room and with participants wearing their daily footwear (39). Participant leg length was measured from the top of the greater trochanter to the ground on both legs. Leg length normalization was used to correct parameters influenced by size and presumably by gender (40). Spatiotemporal Gait Characteristics (SGCs) were recorded in five walking conditions [usual pace (UP), fast pace (FP), slow pace (SP), counting-backwards dual-task (CW) and animal-reciting dual-task (AW)]. Participants were instructed to walk along the walkway, starting two meters before and stopping two meters after the walkway, marked as starting point and end point, respectively, in five different walking conditions. We named this “the 5-Walk test method.” A different instruction was given before each walk. First walk: “Walk from the starting point to the end point at your usual pace like you would walk in the street.” This walk was marked as “usual pace” (UP). Second walk: “Walk from the starting point to the end point as fast as you can without running.” This second walk was marked as “fast pace” (FP). Third walk: “Walk from the starting point to the end point as slow as you can without standing still.” This third walk was marked as “slow pace” (SP). Fourth walk was a dual task: “Walk from the starting point to the end point at your usual speed and count down aloud starting from fifty in steps of two.” This fourth was marked as “counting walk” (CW). Fifth walk was also a dual task: “Walk from the starting point to the end point at your usual speed and name aloud all animals you know.” This fifth walk was marked as “animal walk” (AW). The GAITRite® computer software 4.8.3 Designer automatically calculated the gait speed in centimeters (cm) per second (s), cadence in steps per minute, mean step width in cm, step width variability in percentage, swing time (and cycle time variability in percentage. The gait speed of every walk was normalized according to leg length in meter per second (m/s). The number of steps per meter, a translatable measure of the mean stride length of every walk, was calculated dividing cadence by gait speed or normalized gait speed (steps per meter or normalized steps per meter). Dual-task-cost (DTC, the percentage difference between usual pace and dual-task characteristic) was calculated. The gait characteristics recorded in this study, were listed in a Table S2.

### Exclusion Criteria

Participants were not included when: refusing to participate in standard diagnostic testing, not consenting to the standard procedure for dementia diagnosis, being under age 50 years old, severely demented (MMSE ≤10), unable to walk without help, living in a nursing home and unable to perform the tests due to physical frailty. Participants with orthopedic prostheses or pacemaker patients were excluded from assessment because of interference with the analysis devices.

Participants were excluded from statistical analysis when usual gait speed was too slow (slower than 60 cm/s), swing time variability >30, cycle time variability >1 or Normalized Steps/Meter > 6 at baseline. The participants with slow usual walk were not able to perform the complex tasks in the 5-walk paradigm. This gave rise to missing data on the characteristics in the other four test modalities (fast, slow walking, counting-backwards and animal-reciting while walking), making them less relevant in the search for an overall gait model.

### Statistical Analysis

#### Evolution to a Dementia Over Time

General analysis concerning the final dementia diagnoses per cognitive impairment stage was performed in the total group (>50-years-old), the <70-years-old and the ≥70-years-old group. The percentage of every dementia subtype per stage was reported.

#### The Association Between Quantitative Gait Characteristics in Five Different Walking Conditions and the Dementia Subtypes

A one-way ANOVA was carried out to test if the gait characteristics were different according to the diagnostic outcome, testing the null hypothesis that the gait parameter was equal for all six neurocognitive disorder groups. The analysis was carried out separately for all five tasks (UP, FP and SP, CW and AW), and separately for all five dementia severity groups. During the analysis, we observed that gait characteristics were different between AD and LBD, AD and VascD, but none of the gait characteristics differed between AD and FTD. The difference between LBD and VascD was minimal (Table S3). We simplified the procedure by regrouping the dementia diagnosis outcomes into three subtypes because the study aimed to compare the differentiation capacity of gait characteristics for the eventually dementia subtype diagnosis. The applied umbrella terms for the dementia subtypes were: No Dementia (N), “AD+FTD” dementia subtype and “VascD+LBD” dementia subtype. The “No Dementia” subjects had no dementia diagnosis at baseline and after 5 years of follow-up, and thus remained CDR 0 or CDR 0.5 during the full follow-up period. The “AD+FTD” dementia patients were diagnosed with Alzheimer's dementia or Fronto-temporal lobe dementia within the 5 years follow-up. The “VascD+LBD” dementia patients were those diagnosed with mixed/vascular dementia or Lewy body dementia during follow-up. Due to a large amount of data only CDR 0, 0.5 and 1 are reported. For the gait characteristics per task having a significant *p*-value, a *post-hoc* test was carried out with a Tukey correction for multiple testing. In this *post-hoc* Tukey test, all three diagnostic dementia subtypes were compared to each other and the pairwise differences in mean were given.

#### The Influence of Age on the Association Between Quantitative Gait Characteristics in Five Different Walking Conditions and Dementia Subtypes

To examine the influence of age, we repeated the previous analyses on the dementia subtypes but limited the age range to participants aged years 70 and above (*N* = 374). Compared to the previous analysis, we excluded 59 individuals between ages 50 and 70. All analyses were carried out identically as explained in the previous step.

Due to the multitude of hypotheses tested in this study, using a cut-off of *p* < 0.05 to determine a significant association would lead to an inflated type 1-error. Therefore, associations were tested separately in three CDR groups (CDR 0, CDR 0.5 and CDR 1). These hypothesis tests can be considered independent of each other. Within each CDR group, several gait characteristics were tested, using different tasks and walking speeds. However, the results of these latter tests are not independent, and applying a Bonferroni correction for the total number of hypothesis tests would be overly conservative. Therefore, a cut-off for significance of 0.01 was used here. The data were analyzed using JMP Pro 13.0 (SAS Institute) and the statistical package R version 3.1.2 (Software R by Core Team, 2014).

## Results

### General Descriptive Results

Over a period of 5 years, 536 (61% of 877 eligible) participants were included for the analyses, age ranging from 50 to 95 years old. Clinical inclusion criteria for the study were not met in 342 participants. Additionally, 96 participants were excluded due to a too slow usual gait speed, high cycle time variability or swing time variability or high number of steps/meter. Seven participants had missing data on the diagnosis of the dementia subtype.

**Figure 1:**
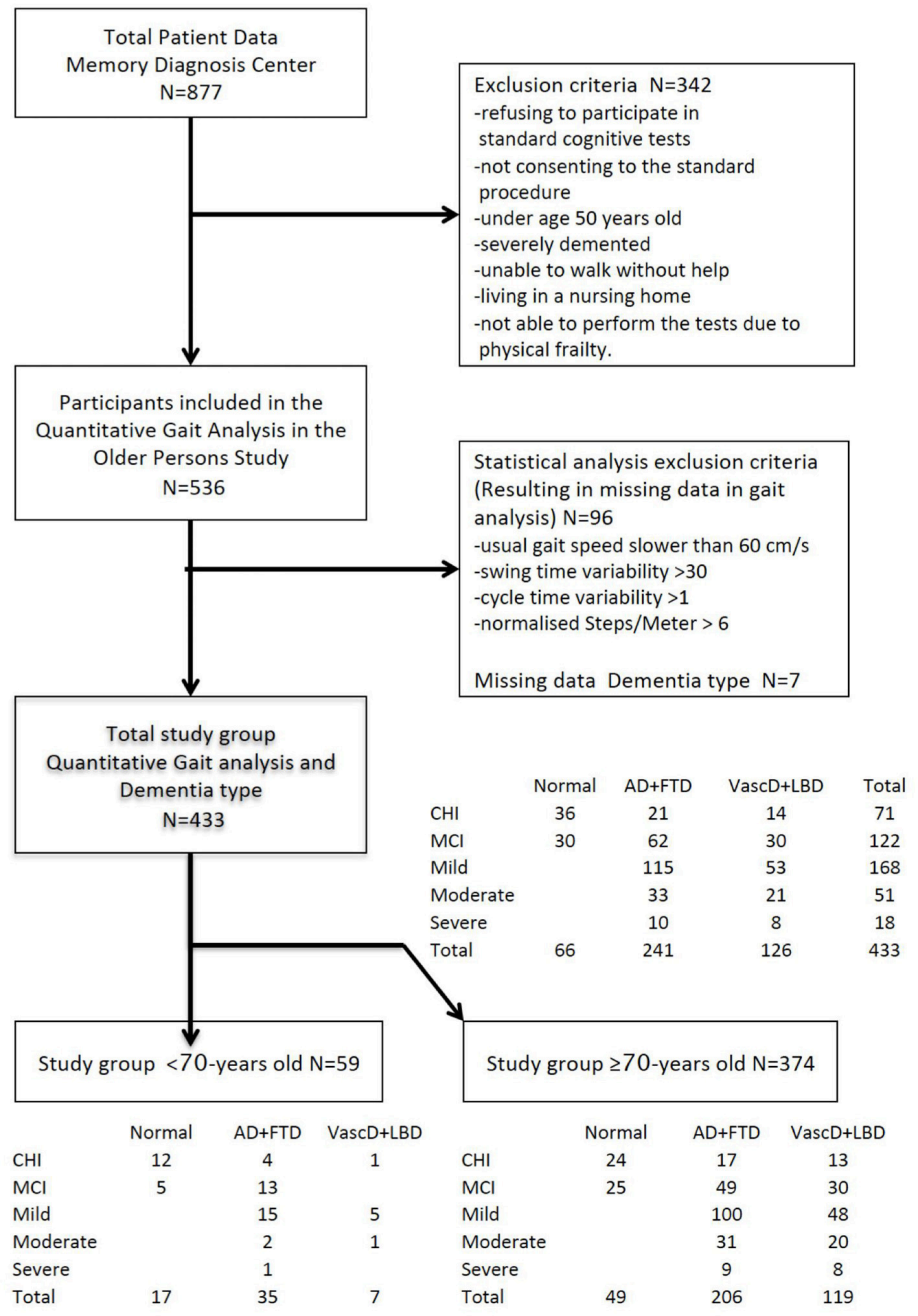
Flowchart of the study. Flow of the selection, categorization, and stratification of the study population. Categories of severity of cognitive decline at baseline according to the CDR scale: CHI, CDR0 cognitively healthy individuals; MCI, CDR0.5 mild cognitively impaired; Mild, CDR1 mildly demented; Moderate, CDR2 moderately demented. Stratification by outcome dementia type at end of follow-up; Normal = no dementia (Cognitively healthy, Mild cognitive impaired), Cortical = cortical dementia (Alzheimer's, Frontal lobe).

Four hundred and thirty-three participants remained for statistical analysis. They were categorized by cognitive impairment stage at inclusion (71 CDR 0, 122 CDR 0.5, 168 CDR 1, 54 CDR 2 and 18 CDR 3). Sub-analysis was performed in the >70 years old group three hundred and seventy-four remained (54 CDR 0, 104 CDR 0.5, 148 CDR 1, 51 CDR 2 and 17 CDR 3). A summary of the patient groups and flow of the analysis is provided in Figure 1.

The results were focused on the gait characteristics for dementia subtyping within the CDR 0, CDR 0.5 and CDR 1 dementia severity groups because these cognitive stages are the most relevant for early detection and differentiation settings. Further, the number of participants in the CDR 2 (*n* = 58) and CDR 3 (*n* = 18) dementia stages was small, and the results for these two groups gave no additional information or insights.

The duration of follow-up ranged from 31 to 41 months. None of the participants reached the full 5 years follow-up. Twelve participants died during the follow-up. Nine of them were CDR 0 to CDR 1 dementia patients. The deceased were all in follow-up for more than 32 months. The mean duration of follow-up was 14 months for those diagnosed “No dementia,” 33 months for participants diagnosed “AD+FTD” and 36 months for those diagnosed “VascD+LBD.”

The time from inclusion to diagnosis in CDR 0 subjects: for “AD+FTD” dementia was 22.0 months (standard error of the mean (SE) = 6.0), and for “VascD+LBD” dementia was 14.0 months (SE = 7.2).

The time from inclusion to diagnosis in CDR 0.5 subjects: for “AD+FTD” dementia was 10.2 months (SE = 2.1), and for “VascD+LBD” dementia was 10.3 months (SE = 2.8).

In the overall group, the final dementia diagnoses per cognitive impairment stage showed an overall evolution of 50% of the CDR 0 group to a dementia stage during the 5 years. Two-thirds of them developed a “AD+FTD” dementia. The rest had a “VascD+LBD” dementia diagnosis. Participants entering the study with CDR 0.5 evolved in 75% of cases to a dementia stage during the study period. Two-thirds of them developed a “AD+FTD” dementia. In the CDR 1 group, 68% of the demented patients had “AD+FTD” dementia at baseline.

**Figure 2:**
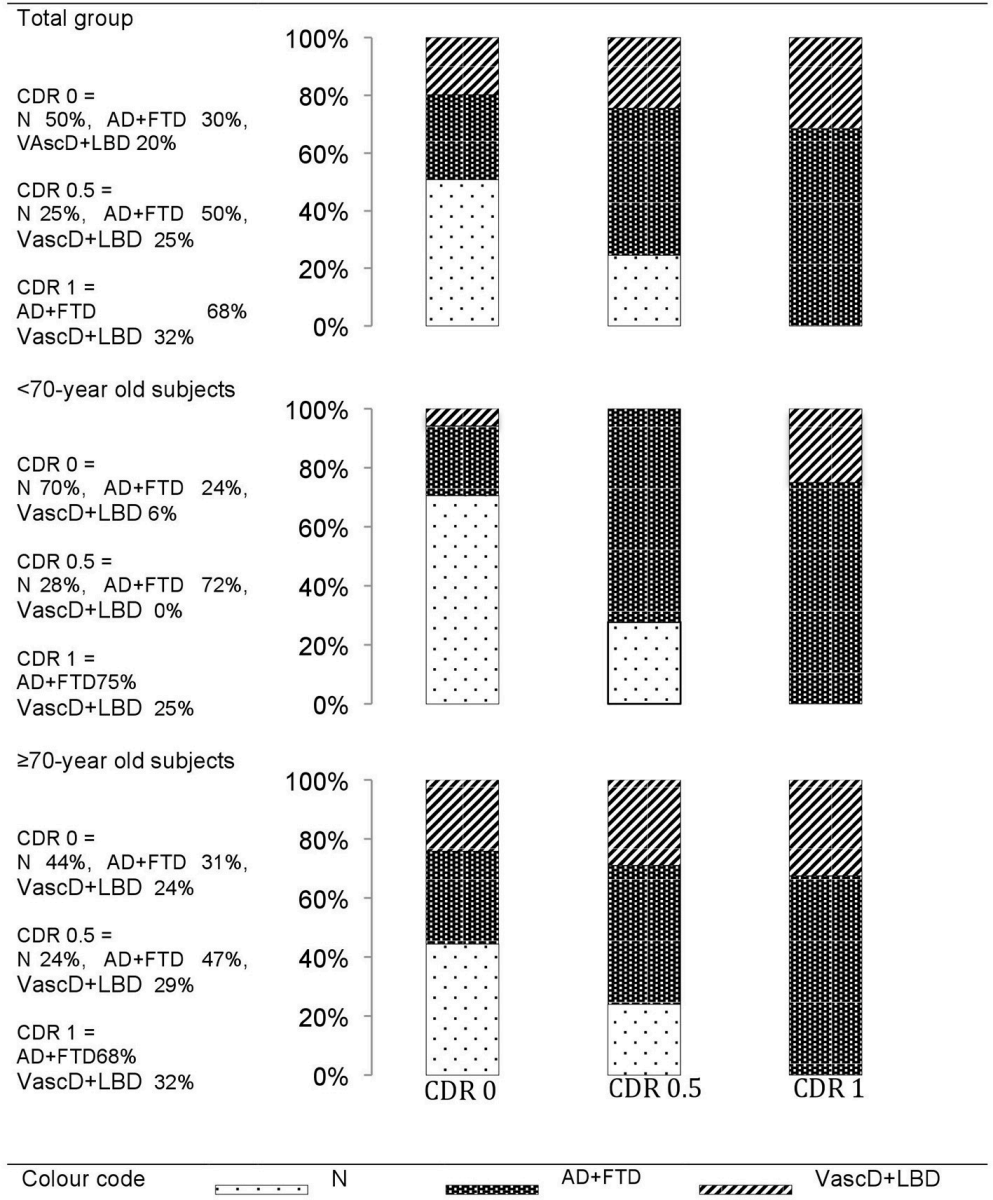
The final dementia diagnoses within the 5-years of follow-up per cognitive impairment stage at baseline. Cognitive impairment stages at baseline: CDR0, cognitively healthy individuals; CDR0.5, mild cognitively impaired; CDR1, mildly demented. Types of Dementia at end of follow-up: N, no dementia (remaining CDR0 or CDR0.5); AD+FTD, grouped Alzheimer's or Frontal lobe dementia; VascD+LBD, grouped Lewy body or vascular dementia.

Only 30% of the <70-years old CDR 0 participants at inclusion evolved toward dementia, whereas 55% of the ≥70-years old CDR 0.5 developed dementia during the study period (Figure 2).

### Differences in Spatiotemporal Gait Characteristics in Five Different Walking Conditions Associate With the Dementia Subtypes in the 50-to-95-Years Old Group

We observed that particular potentially differentiating gait characteristics could be identified per dementia subtype in each cognitive impairment severity stage. An association was suggested between early changes in gait characteristics and the development of a “AD+FTD” or “VascD+LBD” dementia in the near future. The descriptive data and test statistics are reported in Table 1. The significant results of the association tests are in bold. Figure S1 represents the absolute value of the t-ratio of every significant parameter (*p* ≥ 0.01) after *post-hoc* Tukey test, plotted in a histogram.

**Table 1:** Difference in the means of the baseline gait characteristics between outcome dementia diagnosis (AD+FTD/No dementia, VascD+LBD/No dementia and VascD+LBD/AD+FTD dementia type) per cognitive impairment stage in participants >50-years old.

**Cognitive impairment stage at baseline**	**Baseline gait characteristics per dementia type outcome diagnosis (after 3 years of follow-up)**					**Multiple comparisons of means of the gait parameters between dementia types: tukey contrasts**		
**Cognitive stage**	**Gait characteristic**		**No dementia**	**AD+FTD**	**VascD+LBD**	**AD+FTD/No dementia**	**VascD+LBD/No dementia**	**VascD+LBD/AD+FTD**
		**Units**	**Mean (SE)**	**Mean (SE)**	**Mean (SE)**			
CDR 0								
CDR 0.5	UP GS	cm/s	100.9 (3.3)	99.5 (2.3)	92.0 (3.3)		*t*_(62)_ = −3.03, *p* = 0.008	*t*_(90)_ = −3.18, *p* = 0.005
	UP Norm GS	m/s	1.11 (0.03)	1.12 (0.02)	1.02 (0.03)		*t*_(60)_ = −2.92, *p* = 0.01	***t***_**(91)**_ **=** **−3.26**, ***p*** **=** **0.004**
	UP Cadence	Step/min	107.0 (1.6)	106.3 (1.2)	103.1 (1.7)		*t*_(62)_ = −2.62, *p* = 0.02	*t*_(90)_ = −2.56, *p* = 0.03
	UP Norm St/m	Step/m	1.64 (0.05)	1.62 (0.03)	1.72 (0.05)			*t*_(91)_ = 3.04, *p* = 0.008
	FP GS	cm/s	149.7 (5.9)	140.8 (4.1)	133.5 (5.9)		*t*_(62)_ = −2.43, *p* = 0.04	*t*_(91)_ = −2.47, *p* = 0.04
	FP Norm GS	m/s	1.62 (0.06)	1.58 (0.04)	1.49 (0.06)			*t*_(91)_ = −2.59, *p* = 0.02
	FP Norm St/m	Step/m	1.37 (0.04)	1.39 (0.03)	1.45 (0.04)			*t*_(91)_ = 2.85, *p* = 0.01
	CW GS	cm/s	78.0 (4.4)	69.0 (3.1)	66.2 (4.4)		*t*_(62)_ = −2.84, *p* = 0.01	
	CW Norm GS	m/s	0.85 (0.05)	0.77 (0.03)	0.75 (0.05)		*t*_(62)_ = −2.59, *p* = 0.03	
	CW Step width	cm	9.2 (0.6)	8.3 (0.4)	9.2 (0.6)			*t*_(91)_ = 2.48, *p* = 0.04
	CW DTC Step width	%	−35.8 (8.9)	−3.1 (6.3)	−15.8 (8.9)	***t***_**(90)**_ **=** **3.52**, ***p*** **=** **0.002**	*t*_(62)_ = 2.41, *p* = 0.04	
	CW Norm St/m	Step/m	1.77 (0.07)	1.80 (0.05)	1.87 (0.07)		*t*_(62)_ = 2.36, *p* = 0.05	*t*_(89)_ = 2.80, *p* = 0.02
	AW GS	cm/s	77.7 (4.7)	67.3 (3.3)	64.6 (4.7)	*t*_(90)_ = −2.42, *p* = 0.04	*t*_(60)_ = −3.37, *p* = 0.003	
	AW Norm GS	m/s	0.86 (0.05)	0.75 (0.04)	0.73 (0.05)	*t*_(90)_ = −2.59, *p* = 0.03	***t***_**(60)**_ **=** **−3.43**, ***p*** **=** **0.002**	
	AW Step width	cm	8.4 (0.7)	9.0 (0.5)	9.5 (0.7)			*t*_(90)_ = 2.61, *p* = 0.03
	AW Norm St/m	Step/m	1.74 (0.07)	1.88 (0.05)	1.92 (0.07)		*t*_(58)_ = 3.02, *p* = 0.008	
CDR 1	UP Norm GS	m/s		1.05 (0.02)	0.96 (0.03)			*t*_(168)_ = −2.60, *p* = 0.02
	UP Step width	cm		7.8 (0.3)	10.0 (0.4)			***t***_**(170)**_ **=** **4.36**, ***p*** **<** **0.001**
	FP GS	cm/s		133.5 (2.6)	121.2 (3.8)			*t*_(169)_ = −3.07, *p* = 0.006
	FP Norm GS	m/s		1.49 (0.03)	1.35 (0.04)			*t*_(169)_ = −3.26, *p* = 0.003
	FP Cadence	Step/min		127.1 (1.4)	124.5 (2.0)			*t*_(168)_ = −2.56, *p* = 0.02
	FP Step width	cm		7.6 (0.3)	9.1 (0.4)			*t*_(168)_ = 3.13, *p* = 0.005
	FP SwTVar	%		12.1 (0.2)	13.3 (0.3)			*t*_(168)_ = 3.23, *p* = 0.003
	FP Norm St/m	Step/m		1.55 (0.09)	1.58 (0.10			*t*_(167)_ = 2.55, *p* = 0.03
	SP Step width	cm		8.4 (0.3)	11.1 (0.5)			***t***_**(167)**_ **=** **4.74**, ***p*** **<** **0.001**
	SP SwTVar	%		17.9 (0.3)	18.6 (0.4)			*t*_(166)_ = 2.83, *p* = 0.008
	SP Norm St/m	Step/m		2.09 (0.04)	2.26 (0.06)			*t*_(165)_ = 3.05, *p* = 0.006
	CW Step width	cm		8.4 (0.4)	10.9 (0.6)			***t***_**(159)**_ **=** **3.76**, ***p*** **<** **0.001**
	CW DTC St/m	%		−8.9 (1.8)	−18.1 (2.6)			*t*_(159)_ = −3.32, *p* = 0.002
	CW Norm St/m	Step/m		1.86 (0.05)	2.19 (0.07)			*t*_(160)_ = 3.46, *p* = 0.002
	AW Step width	cm		9.4 (0.4)	11.4 (0.6)			*t*_(157)_ = 2.73, *p* = 0.02
	AW DTC St/m	%		−13.0 (1.8)	−19.7 (2.7)			*t*_(157)_ = −2.38, *p* = 0.04
	AW Norm St/m	Step/m		1.95 (0.05)	2.26 (0.07)			*t*_(158)_ = 2.96, *p* = 0.008

*One-way ANOVA paired post-hoc Tukey test (only significant characteristics are reported)*.

*CDR, clinical dementia rating scale; UW, usual pace; FP, fast pace; SP, slow pace; CW, counting walk; AW, animal reciting walk; GS, gait speed; Norm, normalized for leg length; St/m, steps per meter or mean step length; DTC, dual task cost (% difference between parameter in UP and dual task); SwTVar, swing time variability. Descriptive data in Mean [Standard Error (SE)]. Tukey t-test Effect sizes and p-values: t (df) = x, p = a. Statistically Significant values are printed in Bold*.

In the CDR 0 group, no differences in single-task or dual-task gait characteristics were seen between the “no dementia” group and the eventual “AD+FTD” or “VascD+LBD” dementia, nor between the imminent “AD+FTD” and “VascD+LBD” dementia.

In the CDR 0.5 group, however, CW dual-task-cost for step width was larger in the eventual “AD+FTD” dementia participants in comparison to those remaining “no dementia,” and appears to be an important discriminating parameter (*p* = 0.002). Differences between “no dementia” and at-risk for “VascD+LBD” dementia individuals were primarily seen with AW dual tasking gait speed (slower; *p* = 0.003), normalized gait speed (slower; *p* = 0.002) and normalized steps per meter (higher number of steps or shorter step length; *p* = 0.008). The most suggesting gait parameters to indicate differences between imminent “AD+FTD” and “VascD+LBD” dementia seemed the usual pace (UP) gait characteristics. Slower UP gait speed and normalized gait speed and higher normalized steps per meter (*p* = 0.005, 0.004, and 0.008, respectively) in the “VascD+LBD” group showed a difference between the two future dementia subtypes.

In the CDR 1 dementia group, the increasing step width in usual pace (*p* < 0.001), slow pace (*p* < 0.001) and counting walk dual-task (*p* < 0.001) walking condition is suggested to differentiate the most between “AD+FTD” and “VascD+LBD” dementia individuals.

### The Influence of Age

Significant changes in gait characteristics are seen at an earlier stage of cognitive impairment in older adults. The descriptive data and statistics results for the ≥70-years old individuals are reported in Table 2. Figure S2 shows the absolute value of the t-ratio for every significant parameter (*p* ≥ 0.01) after *post-hoc* Tukey test, plotted in a histogram.

**Table 2:** Difference in the means of the baseline gait characteristics between outcome dementia diagnosis (AD+FTD/No dementia, VascD+LBD/No dementia and VascD+LBD/AD+FTD dementia type) per cognitive impairment stage in participants ≥70-years old.

**Cognitive impairment stage at baseline**	**Baseline gait characteristics per dementia type outcome diagnosis (after 3 years of follow-up)**					**Multiple comparisons of means of the gait parameters between dementia types: tukey contrasts**		
**Cognitive stage**	**Gait characteristics**		**No dementia**	**AD+FTD**	**VascD+LBD**	**AD+FTD/No dementia**	**VascD+LBD/No dementia**	**VascD+LBD/AD+FTD**
		**Units**	**Mean (SE)**	**Mean (SE)**	**Mean (SE)**			
CDR 0	UP Step width	cm	6.2 (0.5)	8.3 (0.6)	10.0 (0.7)		*t*_(52)_ = 3.44, *p = 0.004*	
	AW CyTVar	%	0.56 (0.06)	0.69 (0.07)	0.48 (0.08)	***t***_**(51)**_ **=** **−4.19**, ***p=0.003***	***t***_**(51)**_ **=** **−3.76**, ***p=0.007***	
CDR 0.5	UP GS	cm/s	101.0 (3.6)	96.7 (2.6)	92.0 (3.3)		*t*_(104)_ = −3.00, *p =* 0.009	
	UP Norm GS	m/s	1.10 (0.04)	1.09 (0.03)	1.03 (0.03)		*t*_(101)_ = −2.80, *p =* 0.01	*t*_(101)_ = −2.54, *p =* 0.03
	UP Cadence	Steps/min	107.5 (1.8)	106.2 (1.3)	103.1 (1.6)		*t*_(104)_ = −2.58, *p =* 0.03	
	FP GS	cm/s	149.0 (6.5)	136.4 (4.7)	133.5 (5.9)		*t*_(104)_ = −2.55, *p =* 0.03	
	CW GS	cm/s	79.8 (4.8)	66.6 (3.5)	66.2 (4.4)		*t*_(102)_ = −3.16, *p =* 0.006	
	CW DTC Step width	%	*−40.4 (10.1)*	*−5.1 (7.5)*	*−15.8 (9.3)*	***t***_**(102)**_ **=** **3.34**, ***p=*** **0.003**	*t*_(102)_ = 2.50, *p =* 0.03	
	CW Norm St/m	Step/m	1.77 (0.08)	1.85 (0.06)	1.87 (0.07)		*t*_(99)_ = 2.470, *p =* 0.04	
	AW GS	cm/s	*80.5 (4.9)*	*64.8 (3.6)*	*64.6 (4.5)*	*t*_(100)_ = −3.10, *p =* 0.007	***t***_**(100)**_ **=** **−3.77**, ***p<0.001***	
	AW Norm GS	m/s	*0.89 (0.05)*	*0.73 (0.04)*	*0.73 (0.05)*	*t*_(97)_ = 2.59, *p =* 0.03		
	AW Norm St/m	Step/m	1.72 (0.08)	1.92 (0.06)	1.92 (0.07)		*t*_(97)_ = 3.11, *p =* 0.007	
CDR 1	UP GS	cm/s		91.1 (1.7)	83.8 (2.4)			*t*_(148)_ = −2.12, *p =* 0.03
	UP Norm GS	m/s		1.03 (0.02)	0.94 (0.03)			*t*_(146)_ = −2.34, *p =* 0.02
	UP Step width	cm		7.6 (0.3)	10.0 (0.5)			***t***_**(148)**_ **=** **4.16**, ***p*** **<** **0.001**
	UP SwTVar	%		14.8 (0.2)	15.6 (0.3)			*t*_(148)_ = 2.02, *p =* 0.04
	FP GS	cm/s		128.8 (2.5)	119.0 (3.6)			*t*_(148)_ = −2.58, *p =* 0.01
	FP Norm GS	m/s		1.44 (0.03)	1.33 (0.04)			*t*_(147)_ = −2.80, *p =* 0.006
	FP Cadence	Steps/min		126.8 (1.4)	124.4 (2.0)			*t*_(148)_ = −2.44, *p =* 0.01
	FP Step width	cm		7.3 (0.3)	9.1 (0.5)			***t***_**(148)**_ **=** **3.38**, ***p<0.001***
	FP SwTVar	%		12.3 (0.2)	13.5 (0.3)			*t*_(148)_ = 3.11, *p =* 0.002
	SP Step width	cm		8.4 (0.4)	11.2 (0.5)			***t***_**(145)**_ **=** **4.57**, ***p<0.001***
	SP SwTVar	%		18.1 (0.3)	18.9 (0.4)			*t*_(145)_ = 2.63, *p =* 0.02
	SP Norm st/m	Step/m		2.13 (0.04)	2.30 (0.06)			*t*_(145)_ = 2.86, *p =* 0.005
	CW Norm GS	m/s		0.74 (0.02)	0.66 (0.03)			*t*_(140)_ = −2.14, *p =* 0.03
	CW GS	cm/s		66.1 (2.2)	58.7 (3.0)			*t*_(140)_ = −2.04, *p =* 0.04
	CW Step width	cm		8.3 (0.4)	10.9 (0.6)			***t***_**(140)**_ **=** **3.60**, ***p*** **<** **0.001**
	CW DTC St/m	%		−10.2 (2.0)	−18.9 (2.8)			*t*_(141)_ = −2.94, *p =* 0.004
	CW Norm st/m	Step/m		1.90 (0.05)	2.23 (0.07)			*t*_(140)_ = 3.13, *p =* 0.002
	AW Step width	cm		9.5 (0.4)	11.7 (0.6)			*t*_(141)_ = 2.66, *p =* 0.008
	AW DTC St/m	%		−14.2 (2.0)	−20.9 (2.9)			*t*_(138)_ = −2.94, *p =* 0.03
	AW Norm st/m	Step/m		2.00 (0.05)	2.30 (0.08)			*t*_(139)_ = 2.75, *p =* 0.007

*One-way ANOVA paired post-hoc Tukey test (only significant characteristics are reported)*.

*CDR, clinical dementia rating scale; UW, usual pace; FP, fast pace; SP, slow pace; CW, counting walk; AW, animal reciting walk; GS, gait speed; Norm, normalized for leg length; St/m, steps per meter or mean step length; DTC, dual task cost (% difference between parameter in UP and dual task); SwTVar, swing time variability. Descriptive data in Mean [Standard Error (SE)]. Tukey t-test Effect sizes and p-values: t (df) = x, p = a. italic is the universal code for accurate statistical rapporting*.

In the CDR 0 group, the distinction between the “no dementia” and imminent “AD+FTD” dementia group was indicated by a higher AW cycle time variability (*p* = 0.003) in the latter. Discriminating between the “no dementia” and the at-risk for “VascD+LBD” dementia seemed possible with wider UP step width (*p* = 0.004) and higher AW Cycle time variability (*p* = 0.007) in the latter. None of the gait characteristics had significant potential to determine between the two future dementia subtypes.

In the CDR 0.5 older participants, a larger CW dual-task-cost for step width (*p* = 0.003) was suggested to remain the most distinctive gait characteristic between the “no dementia” and the eventual “AD+FTD” dementia group. Also slower AW dual tasking gait speed (*p* < 0.001), normalized gait speed (*p* < 0.001) and higher normalized steps per meter (shorter step length; *p* = 0.007) seemed to persist as differentiating gait characteristics between future “VascD+LBD” dementia participants and those remaining “no dementia.” Not one gait characteristic, on the other hand, had significant differences between the two future dementia subtypes.

The higher step width in usual pace (*p* < 0.001), slow pace (*p* < 0.001) and counting walk dual-task (*p* < 0.001) walking condition remain the most suggestive parameters for differentiation between ≥70-years-old CDR 1 “AD+FTD” and “VascD+LBD” dementia individuals.

## Discussion

This cohort study examined the associations between early changes in gait characteristics and the development of a dementia type in the near future in older adults with cognitive complaints, mild cognitive impairment and mild dementia. The results show that gait characteristics were statistically different across the stages of cognitive impairment and types of dementia. The comprehensive test set-up with five walking conditions also demonstrated multiple significant associations between gait characteristics and the future dementia type. The results were influenced by the age of the participants.

### Gait Characteristics at Different Walking Conditions Differ Dementia Subtype Per Stage of Cognitive Impairment and Emphasize the Value of a Comprehensive Gait Analysis Protocol

Previous research indicated that pace, variability and postural control domain gait characteristics at usual pace were associated with cognitive decline in general and with a difference between Alzheimer and Non-Alzheimer's patients in particular (10, 15, 42, 43). Previous studies also suggests that, already in the pre-dementia stage, changes in gait characteristics during dual-tasking would identify those suffering from a deficient executive function and a higher risk for dementia (35, 44, 45).

In the present study, however, we indicated that the more exhaustive quantitative gait analysis protocol suggested more potentially differentiating gait characteristics per stage of cognitive impairment and identified the discrete signature per dementia type.

The dual-task walking conditions seemed a significant indicators next to usual pace walking conditions to differentiate the cognitively stable from the at risk for “AD+FTD” or “VascD+LBD” dementia in the future, even when cognitive impairment was incipient. In the CDR 0.5 stage, the dual-task postural control domain features (step width) and dual-task pace domain gait features (gait speed and step length) showed an association with future dementia types. Differences in the counting-backwards-dual-task walking test were more indicative for an emergent “AD+FTD” dementia. Changes in Animal-reciting dual-task walking conditions were more linked to an imminent “VascD+LBD” dementia. These results seem to correspond with the experience in neuropsychological testing. This testing makes use of concentration and verbal fluency tasks to differentiate dementia types. The counting-backwards task is seen as a working memory test, more related to frontal lobe dysfunction and to cortical gait changes in early cognitive decline (46). The animal-reciting task is seen as a semantic verbal fluency test relying on semantic memory, knowledge of words and function of the temporal lob. Alzheimer's patients perform better than vascular dementia patients in verbal fluency (47). So far, to our knowledge, none of the dual-task gait characteristics have been determined as more specific indicators for future dementia subtypes. We emphasize that the type of dual-task could be important to expose the risk for a specific dementia subtype. Moreover, the differences were recorded more than 1 year before the dementia diagnosis was made. From this we suggest that motor-cognitive-dual-tasking may be a tool to expose the risk for cognitive decline in a specific dementia subgroup earlier in life. Further research on a larger sample is necessary to define the predictive capacity of these characteristics.

On the other hand, gait characteristics at usual pace seemed the more significant to differentiate between “AD+FTD” and “VascD+LBD” dementia, even in the imminent stage. Gait speed as well as step length were different between the two future dementia types and decreased in the non-Alzheimer group. The difference between “AD+FTD” and “VascD+LBD” dementia in the CDR 1 dementia stage, however, was associated with step width. The relevance of this postural control gait characteristic could indicate that the balance and gait quality evolve differently in the two dementia types. Wider support, step width changes and declining gait quality are related to falls in cognitive impaired, specifically in individuals with white matter lesions and vascular abnormalities in the brain (48–50). The difference was noted in our study in the usual pace test as well as in slow pace or in the counting backwards dual-tasking walking condition indicating that slow walking and distraction during walking might be conditions with a higher risk for falls even in mildly demented patients. This could also indicate that the slow pace test, although considered a single task, introduces an additional complexity for dementia patients with “VascD+LBD” brain damage. The directive “to walk as slow as possible” might be an extra stress factor. Some participants reported during the tests spontaneously that they experienced the slow walking-task as harder to perform. However, qualitative research on this aspect was not performed but could become subject for further analysis in the search for tools in basic screening. Moreover, future research has to focus on the clinical implication of these parameters in differential diagnosis for dementia type and falls risk assessment.

These conclusions underline the added value of a comprehensive gait analysis protocol.

### The Impact of Age on the Differentiation Capacity

Age had an impact on the differentiation capacity of Comprehensive Spatiotemporal Gait Analysis. We observed that differences in gait characteristics appeared at an earlier stage in ≥70-years-old adults. The gait patterns of future “AD+FTD” and “VascD+LBD” dementia patients differed already from the eventually remaining “no dementia” participants at the stage when people were still diagnosed with only memory complaints. Cycle time variability in the animal-reciting dual-task test (variance between time of successive full-step cycles during the dual task test) was suggested to be different in the future “AD+FTD” dementia participants. Animal–reciting dual-task cycle time variability and postural control (step width) at usual pace were found to be indicators for “VascD+LBD” dementia risk. In contrast, the difference between dementia subtypes in older CDR 0.5 patients seemed to disappear. These results suggest that other phenomena, independent of the dementia type and the cognitive impairment stage, might play a role in the changes in motor function in the older adults.

The gait characteristics under consideration (cycle time variability, step width and gait speed) suggest a relationship with differences in executive function, balance and physical performance in the preclinical stage and might correspond with the description of motor cognitive risk (MCR) syndrome (41, 51–53). The relation between early signs of dementia risk and motion were described in this syndrome. However, indirect interpretation of the MCR-syndrome studies suggested also a physical difference. The MRC-prevalence studies profiled patients with diabetes mellitus, chronic obstructive pulmonary disease, obesity and sedentary life at a higher risk for MCR (16, 54). These medical conditions are more susceptible to physical decline. Also, a recent study on the impact of cognitive impairment on the physical aging process described the relation between gait characteristics and relative muscle mass in different cognitive impairment stages (55). From our results we suggest that the aging process might also interfere with the discriminative capacity of gait characteristics between dementia types.

Furthermore, the absence or disappearance of differentiating gait characteristics between “AD+FTD” and “VascD+LBD” dementia types in the CDR 0.5 stage, might also suggest that cognitive and physical aspects related to the gait characteristics become similar in “AD+FTD” and “VascD+LBD” demented older adults. However, this assumption needs to be elaborated. Simultaneous analysis on the gait characteristics, the aging process or frailty and the changes physical parameters like muscle mass, quality and strength in “AD+FTD” and “VascD+LBD” dementia type patients may elucidate the triangular correlation between age, cognition and physical status and the effect of functional decline.

Some limitations of this study need to be addressed. The study was performed in one regional research unit the age, language and ethnical restrictions to an aged Caucasian population could interfere with extrapolation of the results. Dementia differentiation and diagnosis was made only on a clinical basis. The influence of comorbidities was not taken in account. Also the regrouping in “AD+FTD” and “VascD+LBD” dementia types was based on a statistical analysis. This simplification had an impact on the differentiation capacity for a specific dementia type. However, the subgroups were also in specific cases too small to remain a reliable statistical representation for the specific dementia type per cognitive impairment stage. Research in larger datasets will be necessary.

We acknowledge the possibilities of selection bias because the participants were selected after referral for cognitive screening by the general practitioner, a relative or a community social worker. We did not reach persons without cognitive complaints; people living alone without informal or formal care givers, and physically frail persons.

In addition, the exclusion of individuals with pre-existing gait problems, severe physical frailty and severe dementia implied a higher success rate of performing the five walking conditions during multi-testing. However, including these groups would have implied a higher risk of drop out during the five-walk-test. Moreover, due to the restriction to a study group with people walking faster than 60 cm/s at usual pace would have excluded most of them also from statistical analysis. In previous studies, walking too slow was considered related to cognitive decline and had no added value for discriminative power this study aimed for in preclinical to mild dementia.

Furthermore, labeling the at-risk for imminent “AD+FTD” dementia or “VascD+LBD” dementia in a preclinical dementia stage with a gait characteristic was not possible in our general sample of 50-to-95-years old participants. This might be due to the fact that changes in gait develop at a later stage but also that these changes were less important in the 50-to-70-years-old group. We might have excluded the most at risk, the MCR group, from our analyses during the statistical elimination procedure. We left out the individuals with a gait speed at usual pace lower than 60 cm/s (16, 56). Providing a predictive model for dementia subtyping was not possible due to the fact that the number of participants remaining for analysis was too low. Also additional longitudinal analysis might contain the information on gait changes over time. We would make a case for collaborations and analysis in larger data sets to define the place of gait analysis in screening and detection of dementia subtypes.

## Conclusions

Our study aimed to clarify the association between spatiotemporal gait characteristics and dementia typing. The results suggest that specific spatiotemporal gait characteristics are associated with the risk for a specific dementia type in preclinical stage to mild neurocognitive disorders. We conclude that type of cognitive dual-task in combination with the motor task is important. Confounding factors like the aging process and underlying physical status require further research.

## Ethics Statement

Every participant signed an informed consent (IC). The IC was part of the quality procedures maintained in the Memory Diagnosis Centre to assure that patients and their relatives were aware of the diagnostic pathways they were following. For participants with cognitive impairment, the information was given to the patient and the attending informal caregiver, and the IC was obtained from both the patient and the attending informal caregiver. The ethics committee of Emmaus—St. Maarten General Hospital Mechelen approved the study (Emmaus EC 1218).

## Author Contributions

A-MD conceived, planned, designed the experiment, carried out the experiment, the data acquisition, analysis, interpretation, and wrote the manuscript with input from all the authors. EF contributed to statistical data analysis. SP and VV provided critical feedback and proofread the manuscript. OB gave critical advice regarding clinical application and participated in drafting the manuscript and provided a critical review. MV and RR supervised the project. All authors discussed the results and contributed to the final manuscript.

### Conflict of Interest Statement

The authors declare that the research was conducted in the absence of any commercial or financial relationships that could be construed as a potential conflict of interest.
