# Supplementary material for: Comprehensive Quantitative Spatiotemporal Gait Analysis Identifies Gait Characteristics for Early Dementia Subtyping in Community Dwelling Older Adults
Source: Front Neurol. 2019 Apr 5;10:313. doi: 10.3389/fneur.2019.00313 (PMC6459932; doi:10.3389/fneur.2019.00313)
Supplement: Supplementary file 1 [file Table_1.pdf]

Tab S1 *Demographic parameters, Cognitive diagnostic tests and Questionnaires, Physical parameter, Disability assessment scores*

Demographic parameters	Units	Reference
**PERSONAL**		
Age	Years	
Gender	0= female; 1=male	
Ethnicity	Cau= Caucasian, Afr= African, Azi= Asian	
Education	Years	
**MEDICAL HISTORY**		
Depression	0= No; 1= Yes	
Diabetes	0= No; 1= Yes	
Hypertension	0= No; 1= Yes	
Chronic Lung Disease	0= No; 1= Yes	
**COGNITIVE SCORING**		
MMSE	Min = 0; Max= 30	[9]
ACE-R	Min = 0; Max= 100	[22]
NPI-Q	Min = 0; Max= 96	[25]
**FALLS RISK PARAMETERS**		
Fallen in past year	0= No; 1= Yes	
Timed get-up-go (TGUG)	Seconds	[14]
Timed chair stands test (TCST)	Seconds	
Functional reach (FR)	cm	[18]
**NUMBER OF MEDICATIONS**	Discrete number	
**DISABILITY/FRAILTY SCALES**		
Katz score	Min=0; Max=6	[12]
Lawton/Brody score	Min=0; Max= 9	[13]
Rockwood's Clinical Frailty index	Min=0; Max= 9	
