# Supplementary material for: Comprehensive Quantitative Spatiotemporal Gait Analysis Identifies Gait Characteristics for Early Dementia Subtyping in Community Dwelling Older Adults
Source: Front Neurol. 2019 Apr 5;10:313. doi: 10.3389/fneur.2019.00313 (PMC6459932; doi:10.3389/fneur.2019.00313)
Supplement: Supplementary file 2 [file Table_2.pdf]

Tab S2 *Glossary of Quantitative gait analysis characteristics*

Gait characteristics	Units	Description[35]
**GAIT ANALYSIS**		Registration of the characteristics of a gait cycle
**Gait cycle**		(Or stride) anterior-posterior distance between the heel strikes of two successive placements of the same foot
**Gait domains**		
**Single Task (UP, FP, SP) and Dual Task (CW, AW)**		
**Pace domain**		
Gait speed	cm/second	Distance per time unit
Normalised gait speed	M/second	Distance per time unit adjusted for leg length
Cadence	Steps/minute	Number of steps per time unit
Steps/meter	Steps/meter	Number of steps per distance unit
Mean Step length (=steps/meter^{-1})	Meter	Length of one step.
Normalised Steps/meter	Steps/meter	Number of steps per distance unit adjusted for leg length
**Postural control domain**		
Step width	cm	Lateral distance between the midlines of the right and left heels
Step width variability	%	Variance of the Lateral distance between the midlines of the right and left heels between gait cycles
**Variability domain**		
Swing time variability	%	Variance of the time elapsed from the last contact of the current footstep to the first contact of the next footstep on the same foot (between gait cycles)
Cycle time variability	%	Variance of the time elapsed from the first contact of two consecutive footsteps of the same foot (between gait cycles)
**Dual Task Cost (CW, AW)**		Percentage difference between the UP and Dual-task parameter (UPx-DTx)/UPx
Dual task cost Gait speed	%	
Dual task cost Cadence	%	
Dual task cost Step width	%	
Dual task cost Step width variability	%	
Dual task cost Cycle time variability	%	
Dual task cost Swing time variability	%	
Dual task cost Steps/meter	%	
Dual task cost Normalised Steps/meter	%	
**Fluency**		
Number of Counts per 10 meters	Discrete number	
Number of Animals per 10 meters	Discrete number	

Legend: cm: centimetre, M: meter, n: number, UP= Usual pace, FP= Fast pace, SP = slow pace, UPx= Usual pace parameter, DT=dual task (CW =counting walk, AW= animal walk) , DTx= dual task parameter
