# Supplementary material for: Comprehensive Quantitative Spatiotemporal Gait Analysis Identifies Gait Characteristics for Early Dementia Subtyping in Community Dwelling Older Adults
Source: Front Neurol. 2019 Apr 5;10:313. doi: 10.3389/fneur.2019.00313 (PMC6459932; doi:10.3389/fneur.2019.00313)
Supplement: Supplementary file 3 [file Table_3.pdf]

***Tab S3**: Comparison of mean values gait characteristics between 4 dementia types in stage CDR 1 using a Student t-test ((df,n) t-ratio,pvalue*)

Dementia type	AD	FTD	LBD		VascD	
			Gait characteristic	t-test	Gait characteristic	t-test
AD	NS	NS	CWSwingTvar	*t* (1,85) =7.2, p=0.009	UW Norm GS	*t* (1,128) =6.4, p=0.01
			CW St/M	*t* (1,85),=14.7, p<0.001	UW BOS	*t* (1,13) =18.4, p<0.001
			CW N St/M	*t* (1,85) =12.1, p<0.001	UW NSt/M	*t* (1,128) =6.4, p=0.01
			AW St/M	*t* (1,88) =29.6, p<0.001	FW BOS	*t* (1,13) =10.6, p=0.001
			AW DTC St/M	*t* (1,87) =14.7, p<0.001	FW SwingTvar	*t* (1,130) =9.2, p=0.002
			AW NSt/M	*t* (1,88) =16.9, p<0.001	CW BOS	*t* (1,12) =16.6, p<0.001
					CW St/M	*t* (1,124) =8.9, p=0.003
					CW NSt/M	*t* (1,12) =11.0, p=0.001
					AW BOS	*t* (1,124) =8.9, p=0.003
					SW BOS	*t* (1,12) =16.6, p<0.001
FTD	NS	NS	AW NSt/M	*t* (1,16) =11.5, p=0.004	NS	
LBD			NS		AW GS	*t* (1,45) =6.6, p=0.01
					AWCycTvar	*t* (1,45),=8.73 p=0.005
VascD					NS	

Legend: AD : Alzheimer dementia; FTD : Frontotemporal dementia; LBD : Lewy Body dementia; VascD : Vascular dementia ; CDR: clinical dementia rating scale. UW: Usual pace; FP: Fast pace; SP: Slow pace; CW: counting walk; AW: animal reciting walk. GS: Gait speed, Norm : Normalised for leg length, St/m: Steps per meter or mean step length, DTC: Dual task cost (% difference between parameter in UP and dual task), SwTVar: Swing Time Variability. Descripitve data in Mean (Standard Error (SE)). Tukey t test Effect sizes and p-values: *t* (df)=x,*p*=a*)*
