# Supplementary material for: Comprehensive Quantitative Spatiotemporal Gait Analysis Identifies Gait Characteristics for Early Dementia Subtyping in Community Dwelling Older Adults
Source: Front Neurol. 2019 Apr 5;10:313. doi: 10.3389/fneur.2019.00313 (PMC6459932; doi:10.3389/fneur.2019.00313)
Supplement: Supplementary file 5 [file Image_2.pdf]

Fig ≥2 Difference in the means of the baseline gait characteristics between outcome dementia diagnosis (AD+FTD/No dementia, VascD+LBD/No dementia and VascD+LBD/AD+FTD dementia type) per cognitive impairment stage in ≥70-years old participants.

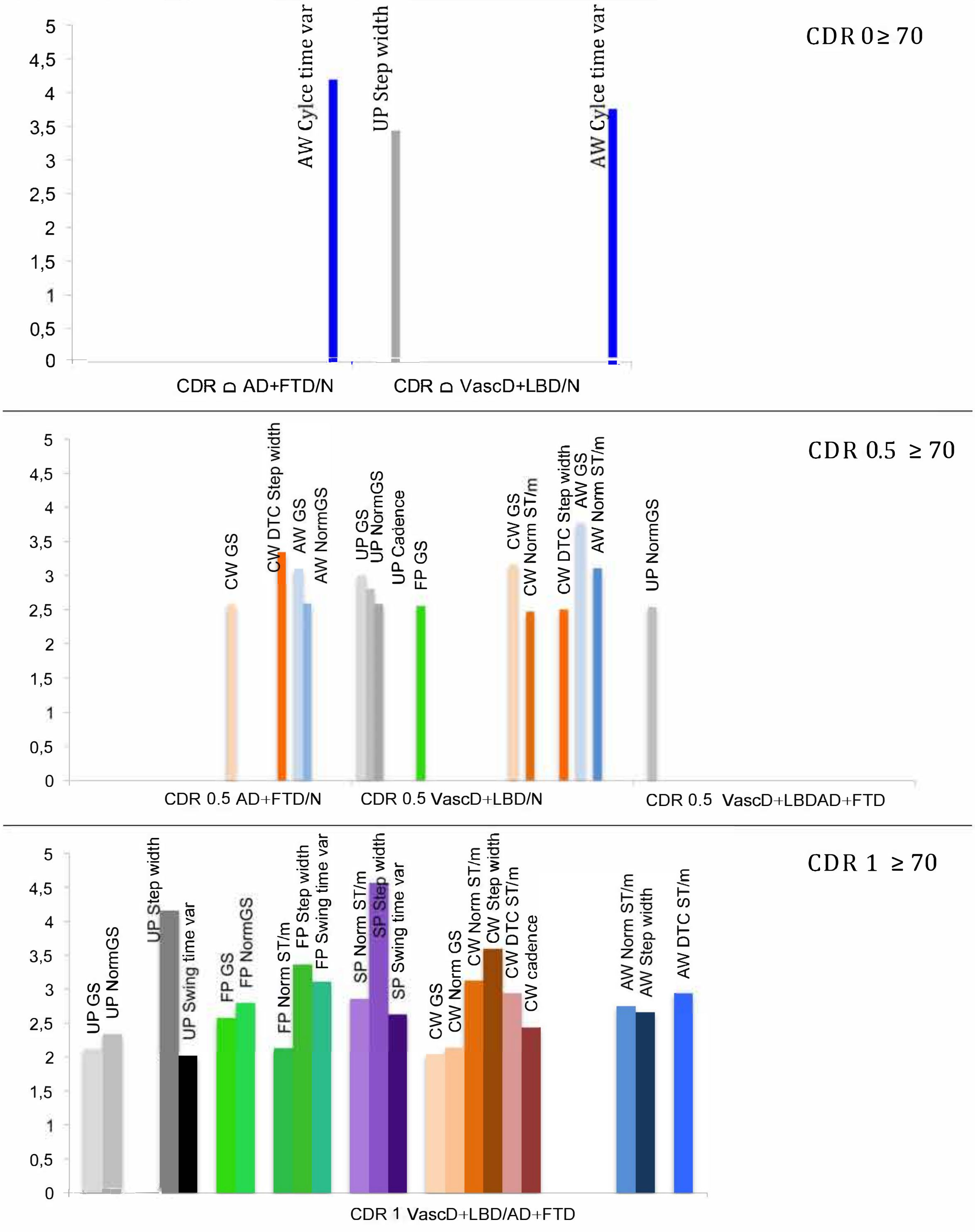

Legend: Colour code: Grey bars UW: Usual pace; Green bars FP: Fast pace; Purple bars SP: Slow pace; Red bars CW: counting walk; Blue bars AW: animal reciting walk. X-axis= Dementia outcome comparison N: C No dementia. Y-axis = t-ratio after paired posthocTukey contrasts t-test

GS: Gait speed, Norm: Normalised for leg length, St/m: Steps per meter or mean step length, DTC: Dual task cost (% difference between parameter in UP and dual task), SwTVar: Swing Time Variability. (Descriptive data in S4.4Table

)
